# Supplementary material for: THLANet: A deep learning framework for predicting TCR-pHLA binding in immunotherapy applications
Source: PLoS Comput Biol. 2025 Sep 12;21(9):e1013050. doi: 10.1371/journal.pcbi.1013050 (PMC12449017; doi:10.1371/journal.pcbi.1013050)
Supplement: S1 Text — (DOCX) [file pcbi.1013050.s001.docx]

THLANet: Deep Learning-Based Prediction of T-Cell Receptor-Antigen Binding Recognition

Long Xu^1^, Qiang Yang^1^, Weihe Dong^1^, Xiaokun Li^1, 2, 9, 10, *^, Kuanquan Wang^1^, Suyu Dong^3*^,

Gongning Luo^1^, Xianyu Zhang^4^, Tiansong Yang^8^, Xin Gao^5, 6, 7, *^, Guohua Wang^1, *^

1School of Computer Science and Technology, Harbin Institute of Technology, Harbin, 150001, China,

2School of Computer Science and Technology, Heilongjiang University, Xuefu Road, Harbin, 150080, China,

3College of Computer and Control Engineering, Northeast Forestry University, Harbin, 150004, China,

4Department of Breast Surgery, Harbin Medical University Cancer Hospital, Harbin 150081, China,

5Computer Science Program, Computer, Electrical and Mathematical Sciences and Engineering Division, King Abdullah University of Science and Technology (KAUST), Thuwal 23955-6900, Kingdom of Saudi Arabia,

^6^Center of Excellence for Smart Health (KCSH), King Abdullah University of Science and Technology (KAUST), Thuwal 23955-6900, Kingdom of Saudi Arabia,

^7^Center of Excellence on Generative AI, King Abdullah University of Science and Technology (KAUST), Thuwal 23955-6900, Kingdom of Saudi Arabia,

^8^Department of Rehabilitation, The First Affiliated Hospital of Heilongjiang University of Traditional Chinese Medicine, Xuefu Road, 150040, Harbin, China

9Postdoctoral Program of Heilongjiang Hengxun Technology Co., Ltd., Xuefu Road, 150090, Harbin, China,

10Shandong Hengxun Technology Co., Ltd., Miaoling Road, 266100, Qingdao, China.

**Section A. HLA pseudo-sequence**

Human leukocyte antigen (HLA) molecules are categorized into HLA class I and class II. HLA class I molecules are ubiquitously expressed on all nucleated cells and primarily present antigens to CD8+ T cells, while HLA class II molecules are selectively expressed on professional antigen-presenting cells to activate CD4+ T cells. This study focuses on HLA class I molecules encoded by three gene loci, which consist of a polymorphic heavy α-chain and a conserved β-2 microglobulin light chain. The α1 and α2 domains of the α-chain form a concave antigen-binding groove through a β-fold structure, enabling peptide binding[^1-3].^

The polymorphic regions of HLA class I alleles are encoded by a pseudo-sequence consisting of 34 amino acid residues that are most likely to interact with peptides. Each residue in the pseudo-sequence is selected based on its proximity to the peptide within 4.0 Å^[4-5]^. These residues reflect the structural diversity of the binding groove, capturing various potential binding conformations. Variations in the polymorphic residues among the HLA-A, -B, and -C alleles are represented through this standardized pseudo-sequence.

To analyze the interaction between peptides and HLA sequences, the frequency of amino acids at the 34 residue positions of the pseudo-sequence was evaluated. Immunogenicity data revealed that the binding grooves of HLA-A, -B, and -C alleles exhibit a relatively consistent pattern in recognizing antigenic peptides. For class I adaptive immune responses, the epitope immunogenicity of pHLA complexes follows a predictable distribution. This regularity supports the application of neural networks to explore the high-level characteristics of immunogenic peptide-HLA interactions^[6]^.

**Section B. Utilizing the CDR3β sequence as the representation for TCR**

T cells play a critical role in immune responses, with most T cells utilizing T-cell receptors (TCRs) composed of α and β chains^[7]^. The primary function of αβ TCRs is to recognize exogenous peptides presented by HLA molecules^[8].^ The key structures involved in antigen recognition are the complementarity-determining region (CDR) loops, which include three loops on the TCRα chain (CDRα1 - CDRα3) and three loops on the TCRβ chain (CDRβ1 - CDRβ3). Due to the highly diverse nature of CDR3β sequences, it is widely accepted that the CDR3β loop plays a central role in recognizing peptide-HLA complexes[14]. Consequently, many computational methods focus solely on CDR3β features to analyze TCR sequences and predict antigen specificity^[11-15]^.

We compiled binding records from datasets such as IEDB^[16]^ and VDJdb^[17]^ and analyzed the distribution of CDR3α, CDR3β, and CDR3(α+β) sequences (as shown Supplementary Table S1-S3, Supplementary Fig. 3). The results revealed that, within these datasets, the number of CDR3 sequences containing α chains or α+β chains is significantly lower than those containing only β chains. This imbalance in data distribution limits the number of samples available for training and testing. Therefore, in this study, we opted to use only CDR3β as the representative feature for TCR sequences. In the future, as more data becomes available, we plan to integrate α chain sequence information into the analytical framework to enhance the comprehensiveness and accuracy of our approach.

**Section C. Data preprocessing for training and testing**

The preprocessing of the training and test datasets was conducted separately to maintain data integrity. For CDR3β sequences, we followed the VDJdb^[17]^ standard by retaining those beginning with the amino acid residue 'C' and ending with 'F'. For HLA sequences, the 34-position encoded pseudo-sequences corresponding to each HLA allele were obtained. Antigens were filtered to include only those with lengths ranging from 7 to 15 amino acids, and duplicate binding pairs were removed. Furthermore, to ensure complete independence, antigen-HLA-TCR triplets that appeared in both the training and test datasets were excluded from the test set.

**Section D. Data curation of HLA-peptide-TCR 3D-crystal complexes**

We sourced HLA-peptide-TCR triplets from the PDB^[18]^ database (https://www.rcsb.org) based on the following criteria: (1) the 3D structure of the complex has been experimentally determined, (2) the peptide is linear, (3) it is presented by HLA class I, and (4) it is from Homo sapiens. Subsequently, we manually curated the CDR3, peptide, and HLA sequences from the PDB, yielding a total of 112 distinct 3D crystal complexes.

**Section E. Data curation of 10X genomics cohort**

The focus of this study is on data obtained from the 10X Genomics Chromium Single Cell Immunoassay Profiling platform, which uses feature barcoding technology to generate single-cell 5' libraries and V(D)J-enriched libraries for TCR sequences identified using highly multiplexed pMHC multimer reagents.

We examined a single-cell dataset containing 44 pMHC complex-unspecific CD8+ T-cell profiles from four healthy donors. These donors included two healthy males aged 18-35 and two healthy females aged 36-50. The dataset was acquired by 10X Genomics from Cellular Technologies Limited.

We analyzed their clonal T-cell expansions to identify TCRs capable of interacting with pMHC complexes. TCRs were quantified based on their original unique molecular identifiers (UMIs). When UMIs > 10, the pMHC-TCR pair was determined to interact with T cells and was counted as a positive sample.

**Section F. Compared methods**

**PanPep**^[20]^ is a robust framework designed for TCR–peptide binding recognition, leveraging meta-learning and a neural Turing machine (NTM) for rapid task adaptation and knowledge retention. It supports few-shot, zero-shot, and majority learning, excelling in generalization and addressing unseen peptides. The model encodes peptide-TCR pairs as 40×5 matrices enriched with sinusoidal positional encoding, processed through a self-attention mechanism and convolutional layers. A peptide-specific learner built via NTM ensures precise mapping between peptide embeddings and task-specific predictors. PanPep demonstrates superior performance in neoantigen recognition, viral immunity, and T-cell repertoire analysis. Its convolutional and attention-based architecture effectively captures contextual features, with dynamic sampling addressing data imbalance. This enables PanPep to deliver highly accurate predictions across diverse datasets, presenting significant potential for clinical applications.

**pMTnet**^[19]^ is a deep learning-based model for predicting TCR–pMHC binding specificity. pMTnet employs stacked autoencoders and LSTM layers to encode TCR CDR3β regions and pMHC sequences, respectively, into low-dimensional embeddings. These embeddings are fused and processed through a fully connected network with dropout and ReLU activation. The model integrates differential loss functions to optimize binding predictions and utilizes transfer learning for handling novel antigens and MHC types. pMTnet significantly outperforms existing methods in benchmark tests, achieving superior ROC and precision-recall metrics. It further analyzes antigen mutation effects on TCR binding and offers critical insights into TCR–pMHC interactions. Demonstrating robust adaptability and precision, pMTnet has potential applications in tumor neoantigen vaccine design, TCR-T therapy optimization, and immune response prediction, making it a valuable tool for clinical immunology research.

**TABR-BERT**^[21]^ is a BERT-based transfer learning model, is designed to predict TCR-pMHC binding specificity with high precision. It comprises two key modules: TCR-BERT and pMHC-BERT, both pre-trained on large-scale unannotated data to address data scarcity. TCR-BERT encodes CDR3β sequences, while pMHC-BERT separately encodes MHC and epitope sequences using segment embeddings. The final TCR-pMHC interaction prediction is achieved through mapping layers and a multi-layer perceptron with contrastive loss for robust distinction between binding and non-binding pairs. TABR-BERT demonstrates superior performance in both zero-shot and non-zero-shot tasks, excelling in generalization to unseen epitopes. Its architecture effectively captures critical sequence features, offering significant potential for applications in neoantigen vaccine design, TCR-T therapy optimization, and personalized immunotherapy.

**References**

1. Wieczorek, M., Abualrous, E. T., Sticht, J., Álvaro-Benito, M., Stolzenberg, S., Noé, F. & Freund, C. Major histocompatibility complex (MHC) class I and MHC class II proteins: conformational plasticity in antigen presentation. Front. Immunol. 8, 292 (2017).

2. Nielsen, M., Lundegaard, C., Blicher, T., Lamberth, K., Harndahl, M., Justesen, S., Røder, G., Peters, B., Sette, A., Lund, O. et al. NetMHCpan, a method for quantitative predictions of peptide binding to any HLA-A and-B locus protein of known sequence. PloS One 2, e796 (2007).

3. Hoof, I., Peters, B., Sidney, J., Pedersen, L. E., Sette, A., Lund, O., Buus, S. & Nielsen, M. NetMHC- pan, a method for MHC class I binding prediction beyond humans. Immunogenetics 61, 1–13 (2009).

4. Reyfhoofnisson, B., Alvarez, B., Paul, S., Peters, B. & Nielsen, M. NetMHCpan-4.1 and NetMHCIIpan-4.0: improved predictions of MHC antigen presentation by concurrent motif de- convolution and integration of MS MHC eluted ligand data. Nucleic Acids Res. 48, W449–W454 (2020).

5. Lu, T., Zhang, Z., Zhu, J., Wang, Y., Jiang, P., Xiao, X., Bernatchez, C., Heymach, J. V., Gibbons, D. L., Wang, J. et al. Deep learning-based prediction of the T cell receptor–antigen binding specificity. Nat. Mach. Intell. 3, 864–875 (2021).

6. Chu, Y., Zhang, Y., Wang, Q., Zhang, L., Wang, X., Wang, Y., Salahub, D. R., Xu, Q., Wang, J., Jiang, X. et al. A transformer-based model to predict peptide-HLA class I binding and optimize mutated peptides for vaccine design. Nat. Mach. Intell. 4, 300–311 (2022).

7. Joglekar, A. V. & Li, G. T cell antigen discovery. Nat. Methods 18, 873–880 (2021).

8. Sidhom, J.-W., Larman, H. B., Pardoll, D. M. & Baras, A. S. DeepTCR is a deep learning framework for revealing sequence concepts within T-cell repertoires. Nat. Commun. 12, 1605 (2021).

9. Valkiers, S., Van Houcke, M., Laukens, K. & Meysman, P. ClusTCR: a python interface for rapid clustering of large sets of CDR3 sequences with unknown antigen specificity. Bioinformatics 37, 4865–4867 (2021).

10. Zhang, H., Liu, L., Zhang, J., Chen, J., Ye, J., Shukla, S., Qiao, J., Zhan, X., Chen, H., Wu, C. J. et al. Investigation of antigen-specific T-cell receptor clusters in human cancers. Clin. Cancer Res. 26, 1359–1371 (2020).

11. Montemurro, A., Schuster, V., Povlsen, H. R., Bentzen, A. K., Jurtz, V., Chronister, W. D., Crinklaw, A., Hadrup, S. R., Winther, O., Peters, B., Jessen, L. E. & Nielsen, M. NetTCR-2.0 enables accurate prediction of TCR-peptide binding by using paired TCR and sequence data. Commun. Biol. 20, 1–13 (2021).

12. Moris, P., De Pauw, J., Postovskaya, A., Gielis, S., De Neuter, N., Bittremieux, W., Ogunjimi, B., Laukens, K. & Meysman, P. Current challenges for unseen-epitope TCR interaction prediction and a new perspective derived from image classification. Briefings Bioinforma. 22, bbaa318 (2021).

13. Springer, I., Besser, H., Tickotsky-Moskovitz, N., Dvorkin, S. & Louzoun, Y. Prediction of specific TCR-peptide binding from large dictionaries of TCR-peptide pairs. Front. Immunol. 11, 1803 (2020).

14. Gao, Y., Gao, Y., Fan, Y., Zhu, C., Wei, Z., Zhou, C., Chuai, G., Chen, Q., Zhang, H. & Liu, Q. Pan-Peptide Meta Learning for T-cell receptor–antigen binding recognition. Nat. Mach. Intell. 5, 235–249 (2023).

15. Peng, X., Lei, Y., Feng, P., Jia, L., Ma, J., Zhao, D. & Zeng, J. Characterizing the interaction conformation between T-cell receptors and epitopes with deep learning. Nat. Mach. Intell. 5, 395–407 (2023).

16. Vita, R., Mahajan, S., Overton, J. A., Dhanda, S. K., Martini, S., Cantrell, J. R., Wheeler, D. K., Sette, A. & Peters, B. The immune epitope database (IEDB): 2018 update. Nucleic Acids Res. 47, D339–D343 (2019).

17. Shugay, M., Bagaev, D. V., Zvyagin, I. V., Vroomans, R. M., Crawford, J. C., Dolton, G., Komech, E. A., Sycheva, A. L., Koneva, A. E., Egorov, E. S. et al. VDJdb: a curated database of T-cell receptor sequences with known antigen specificity. Nucleic Acids Res. 46, D419–D427 (2018).

18. RCSB Protein Data Bank (RCSB.org): delivery of experimentally-determined PDB structures alongside one million computed structure models of proteins from artificial intelligence/machine learning Nucleic Acids Research 51: D488–D508 (2023).

19. Lu, Tianshi, et al. Deep learning-based prediction of the T cell receptor–antigen binding specificity. Nature machine intelligence. 864-875 (2021).

20. Gao, Yicheng, et al. Pan-peptide meta learning for T-cell receptor–antigen binding recognition. Nature Machine Intelligence. 236-249 (2023).

21. Zhang, Jiawei, Wang Ma, and Hui Yao. Accurate TCR-pMHC interaction prediction using a BERT-based transfer learning method. Briefings in Bioinformatics. bbad436 (2024).
