# Supplementary figures and images for: THLANet: A deep learning framework for predicting TCR-pHLA binding in immunotherapy applications
Source: PLoS Comput Biol. 2025 Sep 12;21(9):e1013050. doi: 10.1371/journal.pcbi.1013050 (PMC12449017; doi:10.1371/journal.pcbi.1013050)

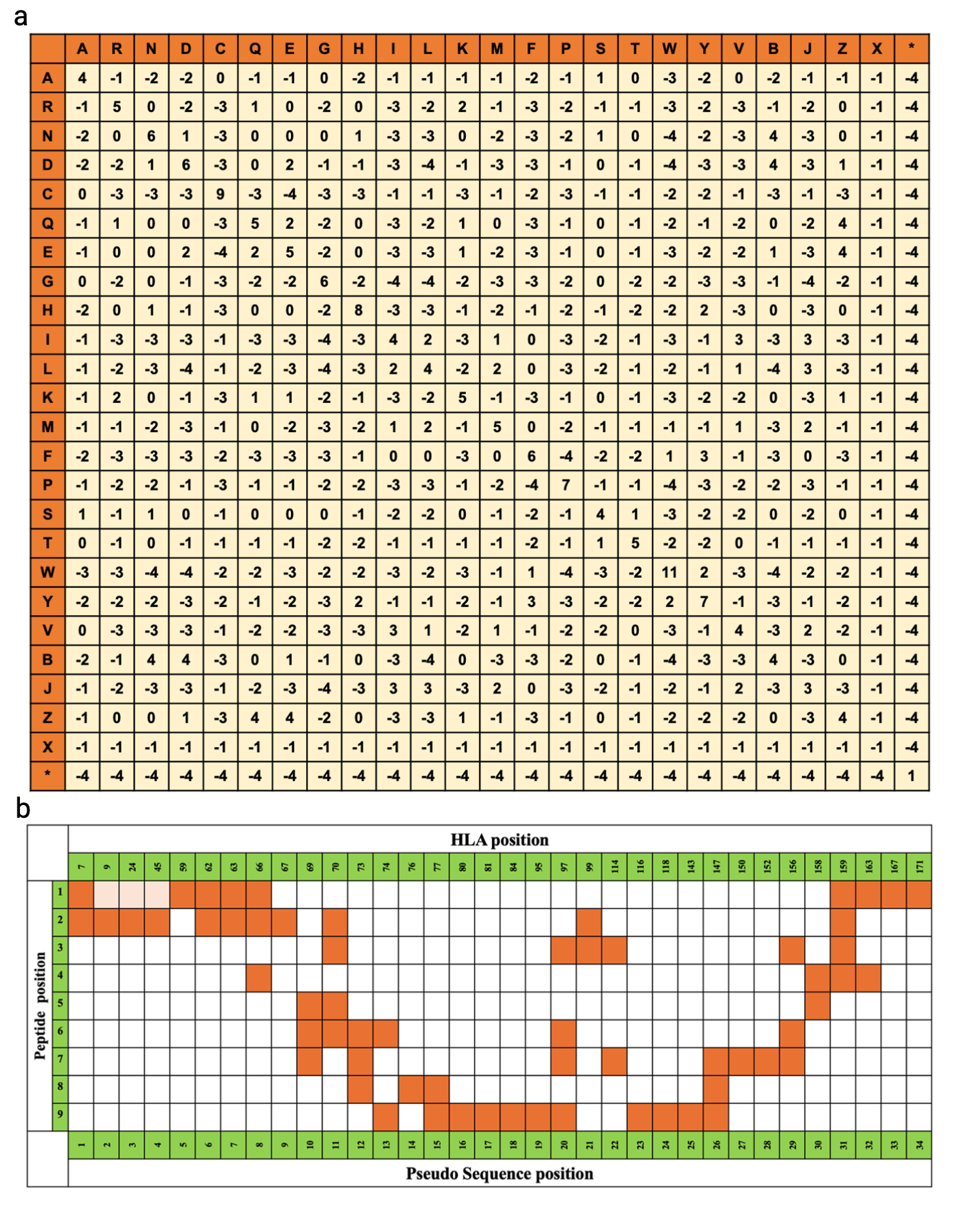

Supplement: S1 Fig — (TIFF) [file pcbi.1013050.s002.tif]

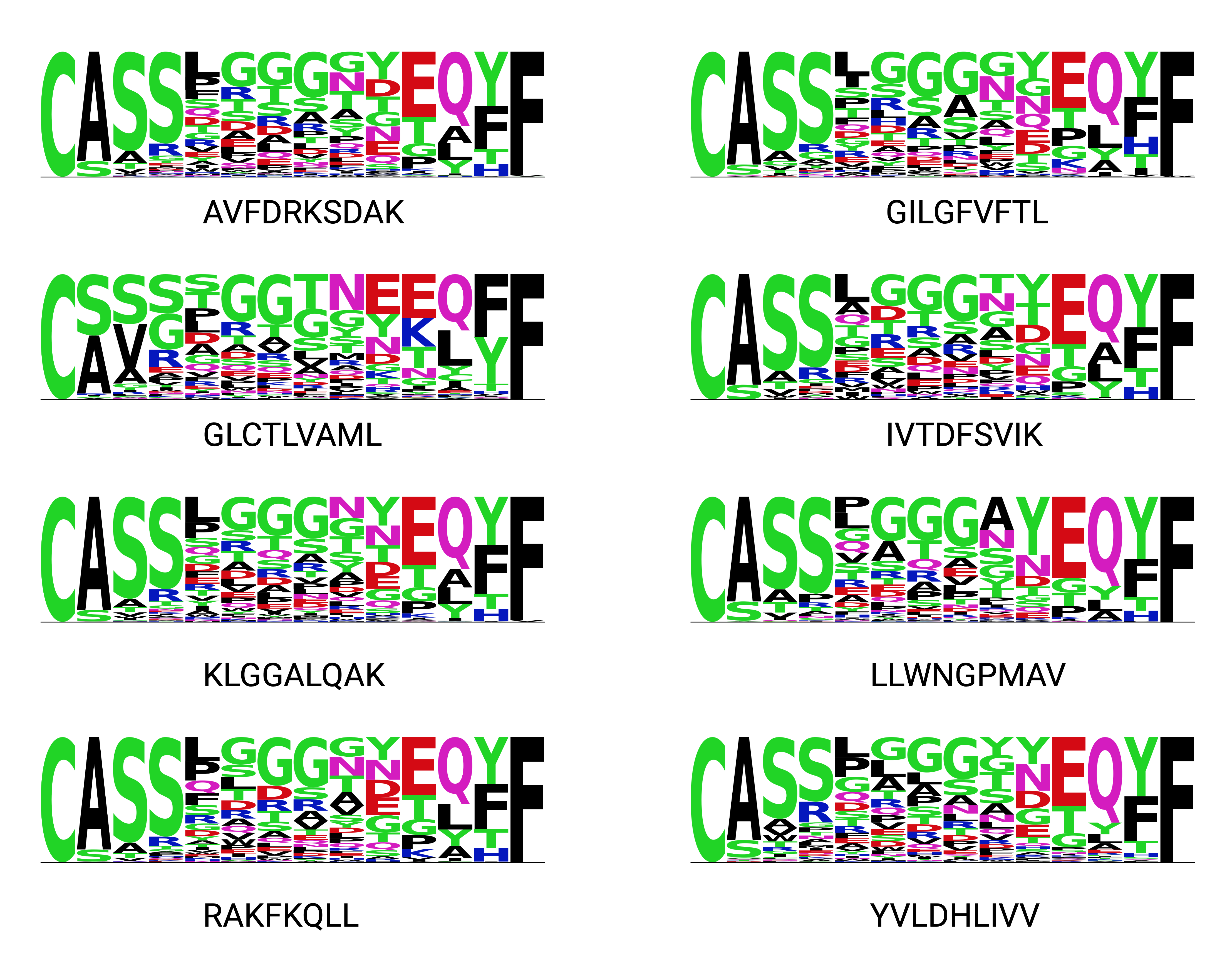

Supplement: S2 Fig — (TIFF) [file pcbi.1013050.s003.tif]

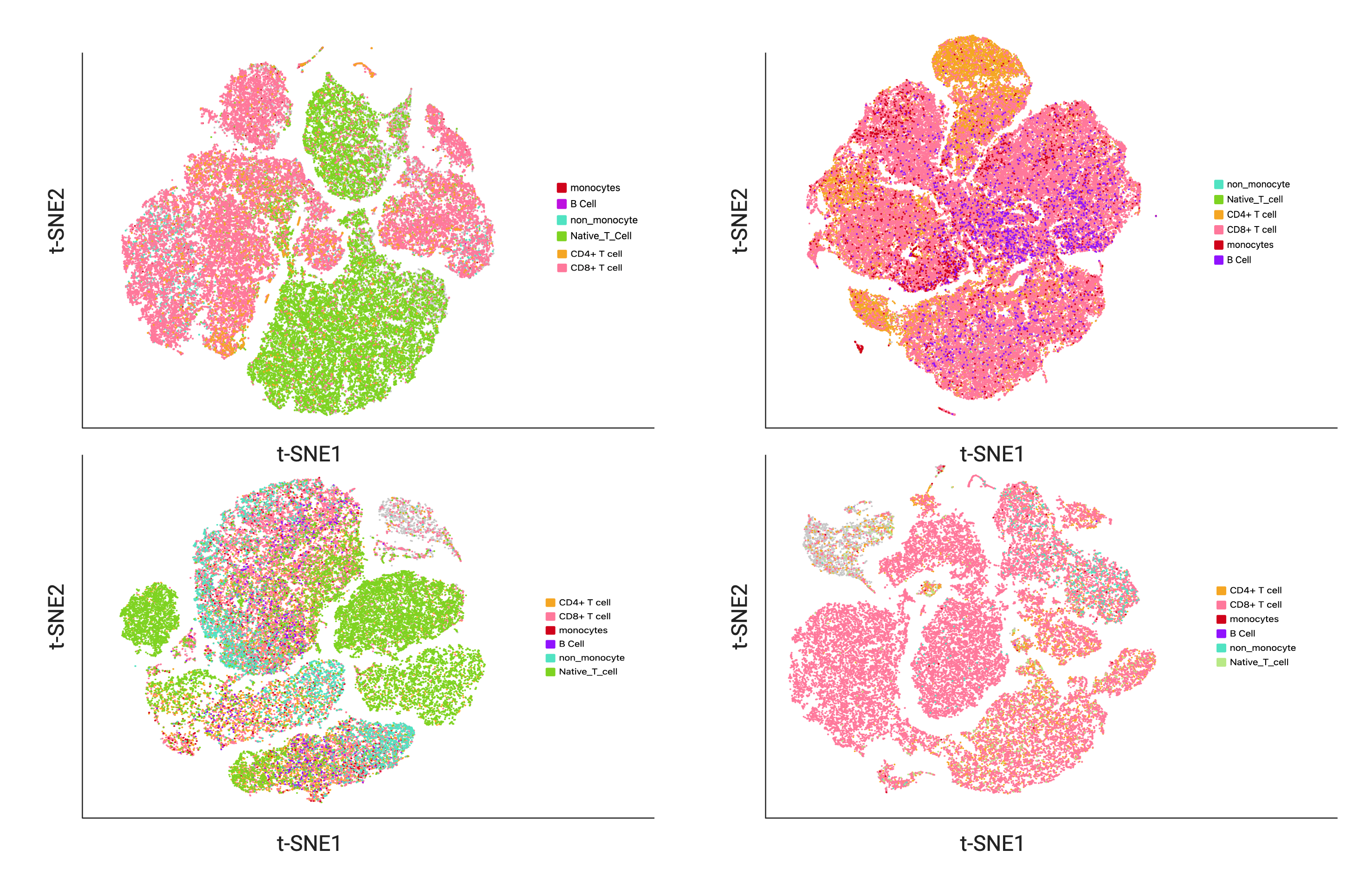

Supplement: S3 Fig — (TIFF) [file pcbi.1013050.s004.tif]

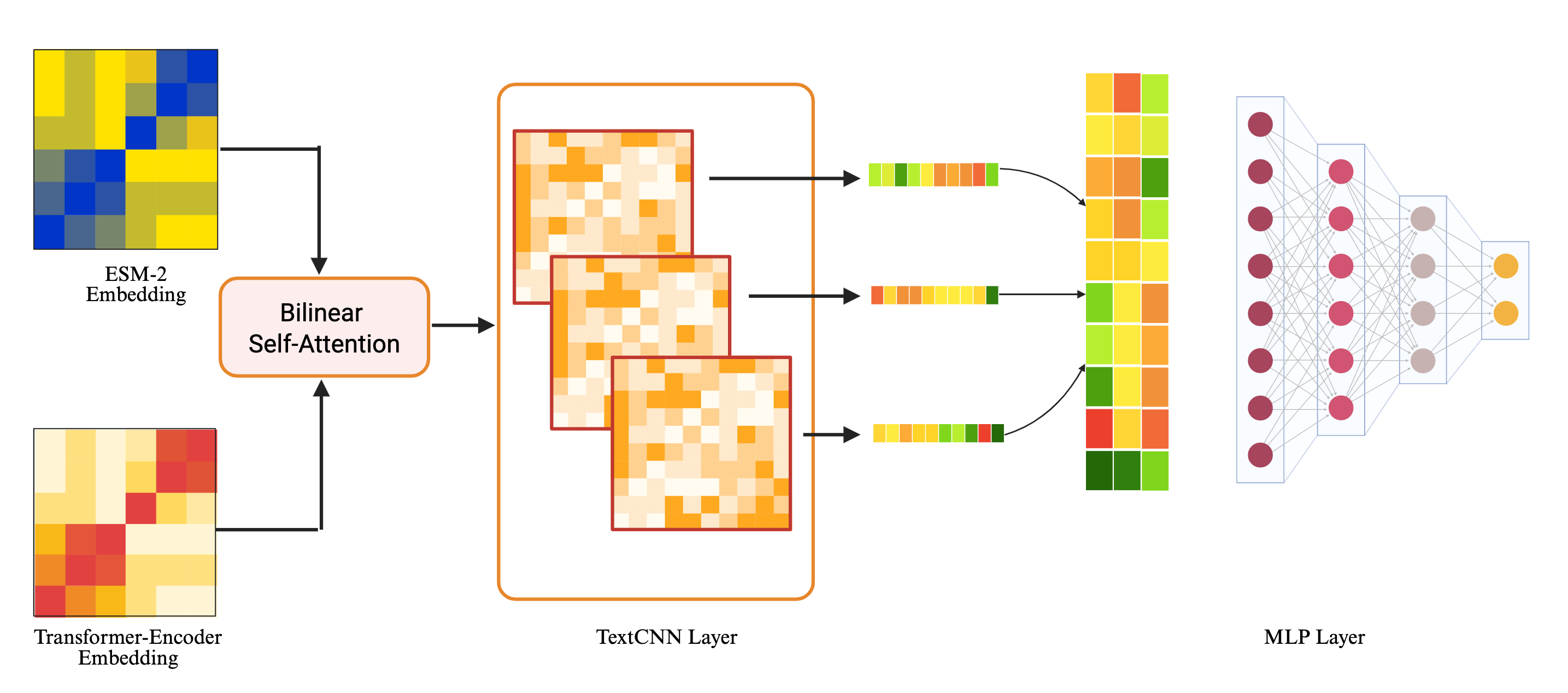

Supplement: S4 Fig — (TIFF) [file pcbi.1013050.s005.tif]

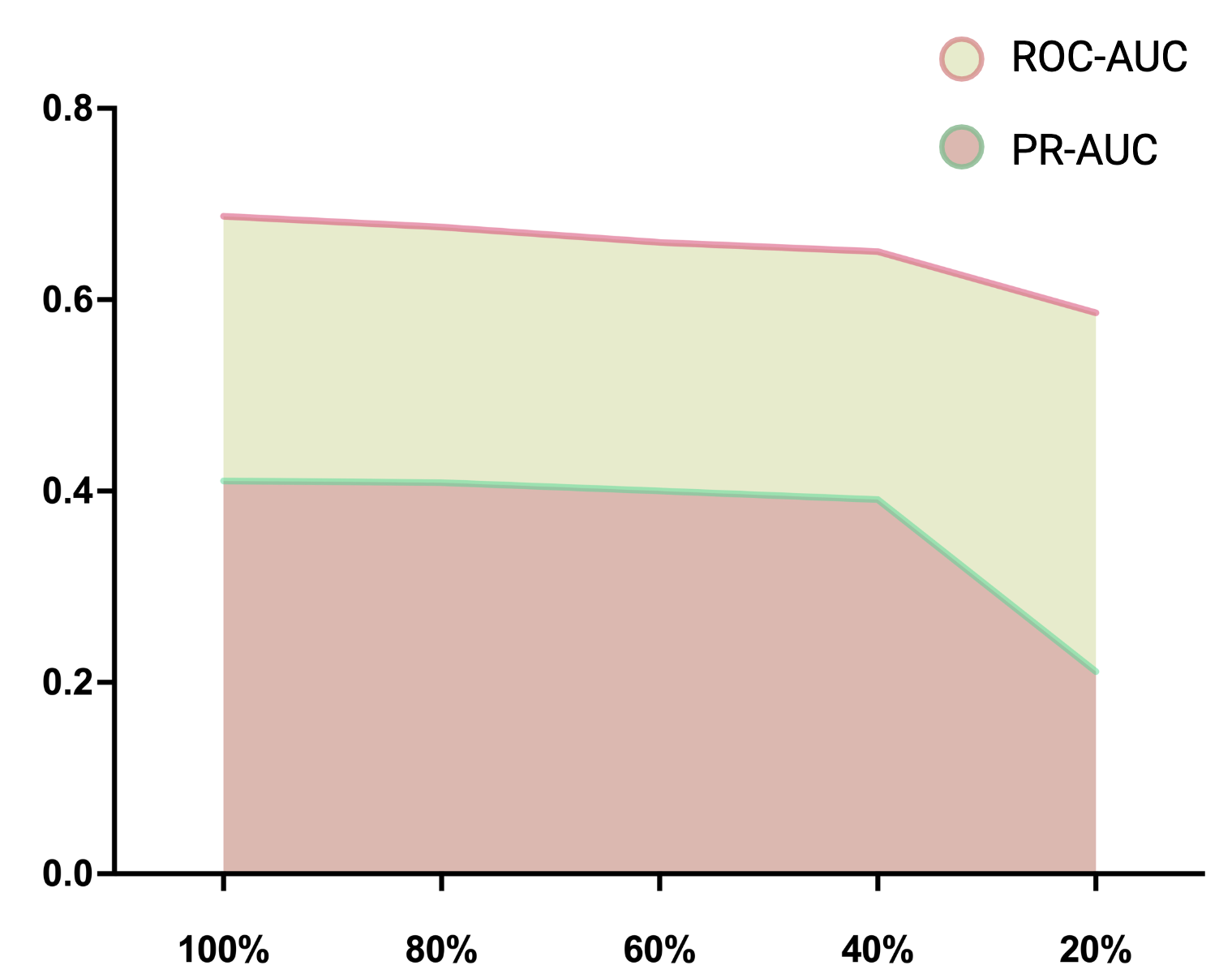

Supplement: S5 Fig — (TIFF) [file pcbi.1013050.s006.tif]
