# Supplementary material for: THLANet: A deep learning framework for predicting TCR-pHLA binding in immunotherapy applications
Source: PLoS Comput Biol. 2025 Sep 12;21(9):e1013050. doi: 10.1371/journal.pcbi.1013050 (PMC12449017; doi:10.1371/journal.pcbi.1013050)
Supplement: Attachment — Submitted filename: Response to Reviewers.pdf [file pcbi.1013050.s019.pdf]

哈爾濱工業大學
HARBIN INSTITUTE OF TECHNOLOGY

No. 92, West Dazhi Street, Nangang District,
Harbin, Heilongjiang, China.
T : 86-0451-86412114
F : 86-0451-86221048
W: www.hit.edu.cn

Dear Dr. Feilim Mac Gabhann, Dr. Inanc Birol, and Reviewers:

We sincerely thank you for your diligent work in coordinating the review process for our manuscript (PCOMPBIOL-D-25-00736). We have carefully reviewed each comment provided by Journal and Reviewers 1, 2, and 3, and we have revised our manuscript to address all suggestions and concerns raised. The feedback has been instrumental in improving the scientific rigor, clarity, and reproducibility of our work.

We are deeply grateful to Reviewers 1, 2, and 3 for their constructive and insightful comments, which have significantly enhanced the quality of our manuscript. We particularly appreciate Reviewer 1's recognition of THLANet's novel insights into TCR-antigen interactions and their detailed suggestions for improving methodological transparency. Reviewer 2's positive remarks on the model's clinical potential and their call for additional details have guided us to strengthen the manuscript's accessibility. Reviewer 3's critical feedback on benchmarking and data dependency prompted us to conduct additional analyses, substantially improving the robustness of our claims. We have strived to maintain the strengths highlighted by the reviewers while addressing their concerns to elevate the manuscript further.

Please find an electronic copy of the revised manuscript and a separate file containing our point-by-point responses to the reviewers' comments. For your and the reviewers' convenience, our responses are presented in blue to clearly distinguish them from the reviewers' original comments.

We hope that you and the reviewers find this revised manuscript acceptable for publication. We look forward to your final decision and appreciate your continued consideration of our work.

Sincerely yours,
Xiaokun Li (on behalf of all the co-authors)

哈爾濱工業大學
HARBIN INSTITUTE OF TECHNOLOGY
No. 92, West Dazhi Street, Nangang District,
Harbin, Heilongjiang, China.
T : 86-0451-86412114
F : 86-0451-86221048
W: www.hit.edu.cn

To dear Reviewer 1:

We are very grateful for your valuable comments on our manuscript. Your insightful suggestions have deepened our research and strengthened our contribution to the field of computational immunology. With your advice, our manuscript has been significantly improved. Our paper introduces THLANet, an innovative deep learning framework that integrates ESM-2 and Transformer-Encoder modules to predict T-cell receptor (TCR) binding to peptide-HLA (pHLA) complexes, advancing cancer immunotherapy research.

Your suggestions, including clarifying preprocessing steps, enhancing reproducibility, and addressing TCR promiscuity, have been critical in refining our work. These points are vital, and we have addressed them comprehensively in the revised manuscript. Below, we provide a detailed point-by-point response to each of your suggestions to ensure clarity and confidence in our revisions. If any aspects remain unclear or require further revision, please contact us immediately. We are committed to further improving the manuscript to meet your expectations.

Major Comments:

1.

Comment:

Considering the complexity of TCR-pHLA interactions and their immunogenicity, what preprocessing steps were applied to the data before training the model? How were the features selected or designed to effectively capture the nuances of these interactions?

Reply:

We sincerely thank the reviewer for their insightful query regarding the preprocessing steps and feature design to address the complexity of TCR-pHLA interactions and their immunogenicity.

To ensure data reliability, we curated high-confidence datasets from validated sources, including 10x Genomics single-cell immunoassays (N=19,657), VDJdb (N=36,168), and IEDB (N=31,836), as described in the ‘Data curation’ section. Duplicates and ambiguous samples were removed, yielding 66,314 unique entries with laboratory-validated positive and negative TCR-pHLA pairs, minimizing noise and ensuring robust representation of immunogenic interactions.

For feature encoding, we employed ESM-2 to capture deep sequence features, supplemented by BLOSUM62 and sinusoidal positional encoding via a Transformer-Encoder to enhance spatial and contextual representation, as detailed in the ‘Data Embedding’ section. A bilinear attention network fused ESM-2 and Transformer-Encoder embeddings, preserving joint features and effectively capturing the nuances of TCR-pHLA interactions.

Please find the corresponding details in the “Data curation” and “Data Embedding” sections in lines “56-164” on pages 4-6.

No. 92, West Dazhi Street, Nangang District,
Harbin, Heilongjiang, China.
T : 86-0451-86412114
F : 86-0451-86221048
W: www.hit.edu.cn

2.

Comment:

Could you provide more details on the experimental setup, including the hardware and software environment? How does the study ensure the reproducibility of its results by the broader scientific community?

Reply:

We sincerely thank the reviewer for their valuable suggestion to provide more details on the experimental setup and ensure reproducibility.

To address this, we have added a comprehensive description of the hardware and software environment used for model training in Supplementary Table S8, detailing the computational resources and software dependencies. Additionally, we have included a detailed summary of model parameters in Supplementary Table S9, outlining the configurations critical for reproducibility. These additions provide clear guidance for the scientific community to replicate our results.

Please find the corresponding details in Supplementary Table S8 and Supplementary Table S9 in the Supplementary Material.

Supplementary Table S8. Software and Hardware Environment

Category	Item	Description
**Software Environment**	Programming Language	Python 3.10
	Computing Framework	CUDA 12.4
	Performance Evaluation and Visualization Tools	scikit-learn v.1.3.0<br>umap-learn v.0.5.5<br>logomaker v.0.8<br>PyMOL v.2.5.8
	Deep Learning Framework	PyTorch 2.7.1
**Hardware Environment**	CPU	Intel(R) Xeon(R) Gold 6248R @ 3.00 GHz, 256 GB RAM
	GPU	2 × NVIDIA A100-PCIE-40GB

No. 92, West Dazhi Street, Nangang District,
Harbin, Heilongjiang, China.
T : 86-0451-86412114
F : 86-0451-86221048
W: www.hit.edu.cn

Supplementary Table S9. Software and Hardware Environment

File	Parameter Category	Parameter Name	Value/Description
**THLAnet**	**Model Parameters**	kernel sizes	[1, 2, 1]
		hidden_dims	[1024, 512, 256]
		dropout_rate	0.3
		Input Shape	[batch_size, 80, 1000]
		Output Shape	[batch_size, 2]
	**Training Parameters**	batch_size	128
		lr	5×10^{-4}
		classification_threshold	0.05
		Optimizer	Adam
		train_split	0.8
		val_split	0.10
		Test Set Proportion	0.10
**Transformer EncoderLayer**	**Model Parameters**	num_layers	3
		d_model	21
		nhead	3
		dim_feedforward	2048
		dropout	0.1
		batch_size	Default 32
**ESM embedding**	**Model Parameters**	Model Architecture	ESM-2 t33, 650M parameters, UR50D pretrained
		Output Shape	[num_samples, 80, 1280]
**ESM2 pretrain**	**Model Parameters**	Model Architecture	ESM-2 650M, 33 layers
		Freeze Strategy	Freeze all parameters, unfreeze first 25 layers
	**Training Parameters**	num_train_epochs	2
		per_device_train_batch_size	2
		learning_rate	1e-5
		save_steps	500
		Optimizer	AdamW

No. 92, West Dazhi Street, Nangang District,
Harbin, Heilongjiang, China.
T : 86-0451-86412114
F : 86-0451-86221048
W: www.hit.edu.cn

3.

Comment:

TCRs are very promiscuous, with a single TCR capable of recognizing more than a million peptides. Do you think this has an impact on the false negatives in the TCR data? What impact does this have on training? Please add some comments to the discussion.

Reply:

We sincerely thank the reviewer for highlighting the important aspect of TCR promiscuity and its potential impact on false negatives and training.

To address this, we have added a concise explanation in the 'Data curation' section, emphasizing TCRs' ability to recognize millions of peptides and the risk of false negatives from random mismatching. We describe our use of laboratory-validated data from 10x Genomics, VDJdb, and IEDB to minimize false negatives, enhancing dataset reliability and THLANet's training accuracy.

Please find the corresponding revision in the section "Data curation" in line "89-94" on page 4.

4.

Comment:

Could you define better what you mean by sinusoidal encoding and why you do it? Readers not familiar with Transformer may not understand.

Reply:

We sincerely thank the reviewers for their detailed suggestions that made the presentation of this paper more concrete and convincing.

We have formulated the principles and applied implications of sinusoidal coding. We used sinusoidal coding to process the vectorized pHLA data more deeply, with the aim of adding positional feature information from the amino acid sequences into the feature matrix, which can be more helpful for the model to understand the relative positions of the amino acids, and to enhance attentional preferences and feature bias between different samples.

The corresponding revision can be found in the section "Data Embedding" in lines "141-143" on pages 6.

No. 92, West Dazhi Street, Nangang District,
Harbin, Heilongjiang, China.
T : 86-0451-86412114
F : 86-0451-86221048
W: www.hit.edu.cn

5.

Comment:

Please consider including your training data in the GitHub repo or providing an accessible link. It can help and guide the readers to repeat your work.

Reply:

We sincerely thank the reviewer for their suggestion to enhance the reproducibility of our work by including training data or providing accessible links.

To address this, we have made the THLANet codebase publicly available on GitHub at https://github.com/ChanganMakeYi/THLAnet. The training data were sourced from validated public databases, including the IEDB (https://www.iedb.org), VDJdb (https://vdjdb.cdr3.net/), and McPAS-TCR (http://friedmanlab.weizmann.ac.il/McPAS-TCR/). Detailed information on the 10x Genomics cohort is accessible at https://www.10xgenomics.com/datasets}. These resources provide comprehensive access to the data, enabling the scientific community to replicate our experiments.

Please find the corresponding details in the “Data Availability” section in lines “495-500” on page 17.

6.

Comment:

Line 57: “We began by analyzing data generated using the 10x Genomics Chromium single-cell immunoassay platform.” Please provide a detailed description explaining how these data were obtained and processed.

Reply:

We sincerely thank the reviewer for this detailed comment and to give us the opportunity to make the presentation of this paper more accurate and proper.

We began by analyzing data generated using the 10x Genomics Chromium single-cell 60 immunoassay platform, which leverages feature barcoding technology to create single-cell 5’ libraries and V(D)J-enriched TCR sequence libraries. This platform also employs highly multiplexed pHLA multimers to identify binding specificity. Specifically, we examined four single-cell datasets obtained from the peripheral blood of four healthy donors with no known viral infections (As shown in Section E in S1 Text). These datasets included 44 CD8+ T-cell profiles that demonstrated specific binding to pHLA complexes. We investigated the clonal expansions of T cells to identify TCRs capable of interacting with pMHC complexes, quantifying them using original unique molecular identifiers (UMIs). When the UMI count reached or exceeded 10, the pMHC-TCR pair was deemed capable of interacting with T cells and was classified as a positive sample. From these single-cell immunoassay datasets, we compiled TCR-pHLA datasets containing 19,657 entries.

Please find the corresponding details in the section “Data curation” in lines “60-72” on page 3.

哈爾濱工業大學
HARBIN INSTITUTE OF TECHNOLOGY

No. 92, West Dazhi Street, Nangang District,
Harbin, Heilongjiang, China.
T : 86-0451-86412114
F : 86-0451-86221048
W: www.hit.edu.cn

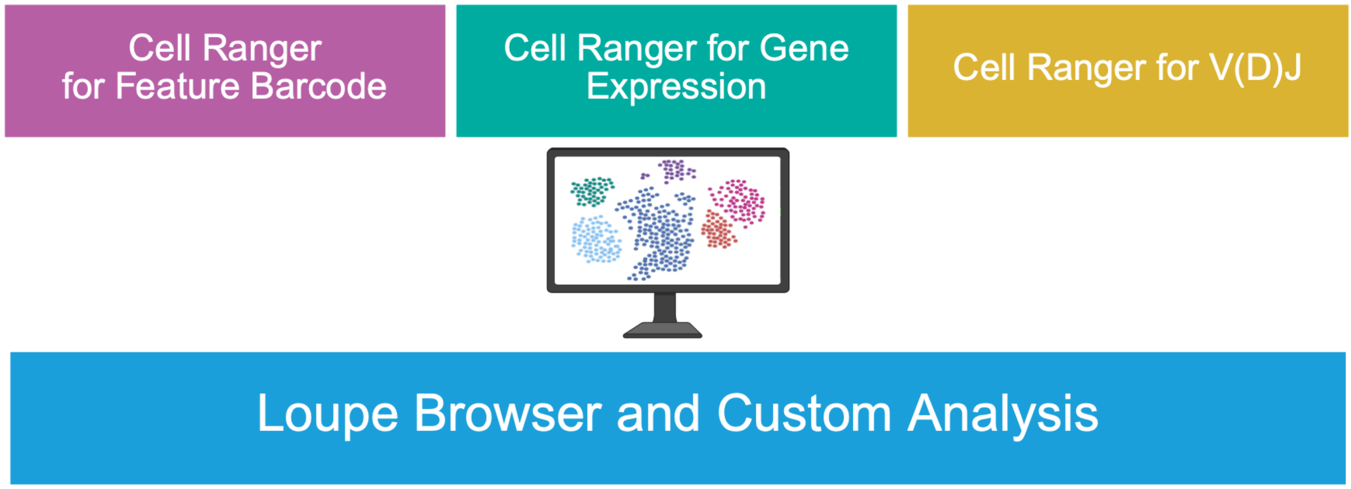

10X Gemomics Data Analysis Tool

7.

Comment:

The manuscript mentions the use of alanine scanning to identify critical binding sites. Why was alanine chosen for simulated amino acid substitutions instead of other amino acids? This may be puzzling for researchers without a biology background; please include an explanation in the text.

Reply:

We sincerely thank the reviewer for their insightful question regarding the choice of alanine scanning, which enhances the clarity of our experimental design.

To address this, we added a paragraph in the “THLANet provides explanation for the spatial structure of the TCR-pHLA interaction” section, explaining the rationale and advantages of alanine scanning. It highlights alanine’s small, non-polar side chain, which minimizes conformational disruption, accurately assesses TCR-pHLA binding contributions, and reduces non-specific interactions due to its prevalence in natural proteins. This revision complements our findings (Fig. 5), reinforcing the method’s validity.

Please find the corresponding revision in the “THLANet provides explanation for the spatial structure of the TCR-pHLA interaction” section, lines “407-411” on page 15.

8.

Comment:

No. 92, West Dazhi Street, Nangang District,
Harbin, Heilongjiang, China.
T : 86-0451-86412114
F : 86-0451-86221048
W: www.hit.edu.cn

The study utilizes ESM-2, a protein language model. To my knowledge, ESM-2 comes in various parameter sizes—why was the 650M parameter version selected? What considerations guided your choice of protein language model parameters?

Reply:

We sincerely thank the reviewer for their insightful question regarding the selection of the ESM-2 model parameters, which enhances the clarity of our model choice.

To address this, we added a paragraph in the "Data Embedding" section, detailing the rationale for selecting the ESM-2 650M parameter version. The ESM-2 650M (33 layers, 650M parameters) balances feature representation depth and computational efficiency for TCR-pHLA binding prediction. Compared to the 150M model, it offers richer sequence features, improving modeling of complex interactions; compared to the 15B model, it requires less memory, suiting our hardware. Pretraining on UniRef ensures robust protein knowledge, suitable for capturing 3D conformational features (Fig. 1b). Experiments confirmed its efficacy with high ROC-AUC and PR-AUC scores (Fig. 3).

Please find the corresponding revision in the "Data Embedding" section, lines "149-158" on page 5.

哈爾濱工業大學
HARBIN INSTITUTE OF TECHNOLOGY

No. 92, West Dazhi Street, Nangang District,
Harbin, Heilongjiang, China.
T : 86-0451-86412114
F : 86-0451-86221048
W: www.hit.edu.cn

To dear Reviewer 2:

We sincerely appreciate your valuable comments on our manuscript. Your suggestions have enriched our study and positioned us to make a more impactful contribution to computational immunology. With your guidance, our manuscript has been substantially improved. Our paper presents THLANet, a novel deep learning framework combining ESM-2 and Transformer-Encoder modules to predict TCR-pHLA interactions, with applications in personalized cancer immunotherapies.

Your recommendations, such as clarifying hyperparameter selection, detailing data splits, and enhancing figure clarity, have been essential in improving the manuscript's rigor and accessibility. We have addressed each of these points in detail in the revised manuscript and provide a comprehensive point-by-point response to your suggestions to ensure transparency and trust in our work. If there are any points that need further clarification or revision, please let us know promptly. We are dedicated to refining the manuscript to fully address your feedback.

Minor Comments:

1.

Comment:

The article mentions the use of the Adam optimizer, a learning rate of 5×10^{-4}, a batch size of 128, etc. (page 7), but does not explain how these hyperparameters were determined.

Reply:

We sincerely thank the reviewer for their valuable comment regarding the determination of hyperparameters, which strengthens the rigor of our experimental design.

To address this, we added a paragraph in the "Experimental setting" section, detailing the hyperparameter selection process. We employed grid search to optimize key hyperparameters, including optimizer (Adam, SGD), learning rate ([1×10^{-4}, 5×10^{-4}, 1×10^{-3}]), batch size ([64, 128, 256]), and weight decay ([1×10^{-5}, 1×10^{-4}]). Model performance was evaluated on the validation set using ROC-AUC as the primary metric, identifying Adam (for adaptive gradient adjustment), a learning rate of 5×10^{-4} (balancing convergence and stability), and a batch size of 128 (suitable for our NVIDIA A100-PCIE-40GB hardware and gradient stability) as optimal. These hyperparameters, combined with early stopping, achieved high ROC-AUC and PR-AUC scores (Fig. 3), confirming their suitability.

Please find the corresponding revision in the "Experimental setting" section, lines "244-255" on page8.

No. 92, West Dazhi Street, Nangang District,
Harbin, Heilongjiang, China.
T : 86-0451-86412114
F : 86-0451-86221048
W: www.hit.edu.cn

2.

Comment:

How did you split the data into training and validation? Did you ensure no overlap or closely similar sequences between TCRs or antigen sequences?

Reply:

We sincerely thank the reviewer for their insightful questions regarding data splitting and sequence similarity, which enhance the transparency of our data processing.

To address this, we added a paragraph in the "Data curation" section detailing the data splitting and sequence similarity control methods. The dataset was randomly split into training, validation, and test sets in an 8:1:1 ratio, ensuring sample independence. This approach minimized data leakage, enhancing model generalizability.

We confirmed that the dataset contains no duplicate samples, ensuring no risk of data leakage. However, the presence of similar sequences in T-cell receptors (TCRs) is unavoidable, as TCR sequences are derived from the transcription and translation of genes at fixed genomic loci.

Please find the corresponding revision in the "Pseudosequence Construction and Data Preparation, lines "183-185" on page 7,

3.

Comment:

How did you get the test dataset in Section 3.1?

Reply:

We sincerely thank the reviewer for their query regarding the source of the test dataset, which enhances the transparency of our data selection.

To address this, we added a section "Pseudosequence Construction and Data Preparation", detailing the test dataset origins. The test dataset for Fig. 3a was derived from 10% of the main dataset (N=66,314), randomly split in an 8:1:1 ratio, comprising TCR-pHLA pairs from 10x Genomics(N=19,657), VDJdb (N=36,168), and IEDB (N=31,836).

Please find the corresponding revision in the "Data curation" section, lines "73-88" on page 6.

4.

Comment:

Where did the "peer-reviewed data from melanoma and gastrointestinal cancers" come from? Please cite the related references.

No. 92, West Dazhi Street, Nangang District,
Harbin, Heilongjiang, China.
T : 86-0451-86412114
F : 86-0451-86221048
W: www.hit.edu.cn

Reply:

We sincerely thank the reviewer for their query regarding the source of the "peer-reviewed data from melanoma and gastrointestinal cancers."

Fig. 3c datasets were sourced from Stevanović et al. (2017) [44] (melanoma antigens) and Tran E, et al. (2015) [45] (gastrointestinal cancer somatic mutation antigens). Fig. 3d dataset was obtained from bone marrow cells of a T-cell prolymphocytic leukemia (T-PLL) patient (Discovery Life Sciences, 10x Genomics), labeled with 44 dCODE™ Dextramer® reagents (Immudex) and TotalSeq™-C antibodies (BioLegend), with Dextramer^{+} T-cells enriched via FACS (CG000203). All datasets underwent deduplication and validation, ensuring sequence independence. Results (Fig. 3) showed high ROC-AUC and PR-AUC, confirming their reliability.

Please find the corresponding revision in the "Data curation" section, lines "116-120" on page 4.

5.

Comment:

The conclusion of Section 3.4 that the central part of CDR3 exhibited higher importance in TCR-pHLA interactions is a well-known notion.

Reply:

We thank the reviewer for their comment on the conclusion of Section "THLANet provides explanation for the spatial structure of the TCR-pHLA interaction".

We added a paragraph, acknowledging the known importance of CDR3's central region, the importance of the CDR3 central region in TCR-pHLA interactions is well-recognized. However, THLANet provides novel quantitative evidence through computational alanine scanning, precisely identifying key residues impacting binding affinity. Analysis of 112 high-quality TCR-pHLA 3D structures revealed spatial interaction patterns, offering new insights into binding mechanisms, complementing existing knowledge, and supporting immunotherapy applications.

Please find the corresponding revision in the "THLANet provides explanation for the spatial structure of the TCR-pHLA interaction" section, lines "435-438" on page 15.

6.

Comment:

It would be better to add a figure to depict the last MLP module of Figure 2b.

Reply:

We sincerely thank the reviewer for their valuable suggestion to include a figure to better illustrate the

No. 92, West Dazhi Street, Nangang District,
Harbin, Heilongjiang, China.
T : 86-0451-86412114
F : 86-0451-86221048
W: www.hit.edu.cn

last multilayer perceptron (MLP) module in Figure 2b.

To address this, we have added Supplementary Fig. 5, titled "The training model structure of THLANet," to the supplementary materials. This figure provides a comprehensive schematic of the THLANet training model, detailing the architecture, including the final MLP module, and clearly illustrating its structure and data flow. This complements Figure 2b, enhancing readers' understanding of the model's implementation details and the prediction process for TCR-pHLA interactions.

Please find the corresponding revision in Supplementary Fig. 5 in the supplementary materials.

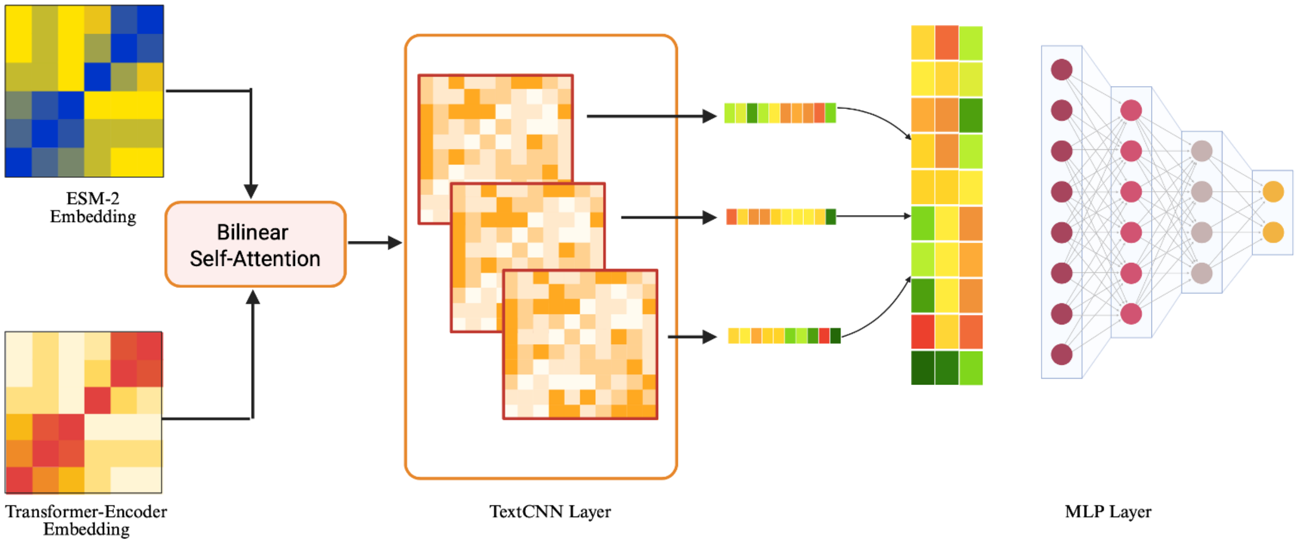

Supplementary Fig. 5. The training model structure of THLAnet.

7.

Comment:

The input to ESM-2 and to Transformer-Encoder model were not described explicitly, and some details need to be added.

Reply:

We sincerely thank the reviewer for their comment on the input descriptions for ESM-2 and Transformer-Encoder, which enhances the transparency of our methodology.

To address this, we added a paragraph in the "THLANet architecture" section, clarifying the input details. ESM-2 receives standardized protein sequences (antigen: 15 residues, CDR3β: 25 residues, HLA: 34 residues), padded with <pad>, and appended with <cls> and <eos> tokens, yielding an [n, 80, 1280] feature matrix. The Transformer-Encoder takes BLOSUM62-encoded TCR-pHLA sequences (80×21

matrix) with sinusoidal positional encoding. Detailed parameters are provided in Supplementary Table S9. This complements existing output structure descriptions, ensuring clarity. Results (Fig. 3) validate the effectiveness of these input designs.

Please find the corresponding revision in the "THLANet architecture" section, lines "186-203" on page 7, and Table S9 in Supplementary Material.

Supplementary Table S9. Software and Hardware Environment

File	Parameter Category	Parameter Name	Value/Description
THLAnet	Model Parameters	kernel sizes	[1, 2, 1]
		hidden_dims	[1024, 512, 256]
		dropout_rate	0.3
		Input Shape	[batch_size, 80, 1000]
		Output Shape	[batch_size, 2]
	Training Parameters	batch_size	128
		lr	5×10^{-4}
		classification_threshold	0.05
		Optimizer	Adam
		train_split	0.8
		val_split	0.10
		Test Set Proportion	0.10
Transformer EncoderLayer	Model Parameters	num_layers	3
		d_model	21
		nhead	3
		dim_feedforward	2048
		dropout	0.1
		batch_size	Default 32
ESM embedding	Model Parameters	Model Architecture	ESM-2 t33, 650M parameters, UR50D pretrained
		Output Shape	[num_samples, 80, 1280]
ESM2 pretrain	Model Parameters	Model Architecture	ESM-2 650M, 33 layers
		Freeze Strategy	Freeze all parameters, unfreeze first 25 layers
	Training Parameters	num_train_epochs	2
		per_device_train_batch_size	2
		learning_rate	1e-5
		save_steps	500
		Optimizer	AdamW

No. 92, West Dazhi Street, Nangang District,
Harbin, Heilongjiang, China.
T : 86-0451-86412114
F : 86-0451-86221048
W: www.hit.edu.cn

To dear Reviewer 3:

We are deeply grateful for your insightful and valuable comments on our manuscript. Your critical feedback has significantly advanced our research and enhanced our contribution to immunoinformatics. With your help, our manuscript has undergone substantial improvements. Our paper introduces THLANet, a cutting-edge deep learning framework that leverages ESM-2 and Transformer-Encoder architectures to predict TCR-pHLA binding, supporting advancements in cancer immunotherapy.

Your suggestions, particularly regarding benchmarking, data dependency, and the appropriateness of ESM-2, have been pivotal in strengthening the manuscript's scientific rigor and generalizability. We have carefully addressed each point in the revised manuscript and provide a detailed point-by-point response to your comments to ensure clarity and confidence in our revisions. If any aspects require further explanation or modification, please contact us immediately. We are fully committed to improving the manuscript to meet your standards.

Thank you for your rigorous and thoughtful feedback.

Major Comments:

1.

Comment:

The choice of ESM-2 as a general protein embedding tool raises concerns about its appropriateness for describing TCR–pMHC interactions. Given that ESM-2 is not specialised for immune recognition, the manuscript should explicitly demonstrate that ESM-2 embeddings effectively separate or distinguish TCR and pMHC sequences in embedding space. Alternatively, developing or employing a language model specifically trained for TCR–pMHC data would strengthen confidence in the model's predictive capabilities.

Reply:

We appreciate the reviewer's suggestion. To address this, we developed a specialized language model by fine-tuning ESM-2 (650M) on a curated dataset of TCR CDR3β, HLA, and antigen sequences tailored for immune-related interactions. This fine-tuned model was optimized using masked language modeling to enhance its ability to capture high-dimensional features specific to TCR–pMHC interactions.

Retraining THLANet with embeddings from the fine-tuned model significantly improved performance. On the test dataset, THLANet achieved a median ROC-AUC of 0.8905 (vs. 0.8803 for original ESM-2) and PR-AUC of 0.8587 (vs. 0.8552) (Fig. 3a). In clinical datasets, it improved ROC-AUC to 0.8371 (vs. 0.8152) and PR-AUC to 0.7223 (vs. 0.6845) for melanoma and gastrointestinal cancers, and ROC-AUC to 0.7142 (vs. 0.6976) and PR-AUC to 0.7192 (vs. 0.7170) for T-PLL (Fig. 3c-d), with all improvements statistically significant (two-tailed Wilcoxon signed-rank test, $p<0.05$). UMAP analysis of the fine-tuned embeddings (Fig. 1b) showed tighter clustering of TCR, HLA, and antigen sequences, indicating richer

No. 92, West Dazhi Street, Nangang District,
Harbin, Heilongjiang, China.
T : 86-0451-86412114
F : 86-0451-86221048
W: www.hit.edu.cn

feature representations.

These results, now incorporated in the revised manuscript, confirm that the fine-tuned model enhances THLANet's predictive accuracy and generalization for TCR–pMHC interactions, bolstering confidence in its clinical applicability. The relevant data and pre-trained model code have been made available on GitHub (https://github.com/ChanganMakeYi/THLAnet).

Please find the corresponding revision in the "Data Embedding" section, lines "136-139" on page 6, the "THLANet architecture" section, lines "186-201" on page 7, "Fig. 1b" on page 5, the Table S9 in the supplementary Material.

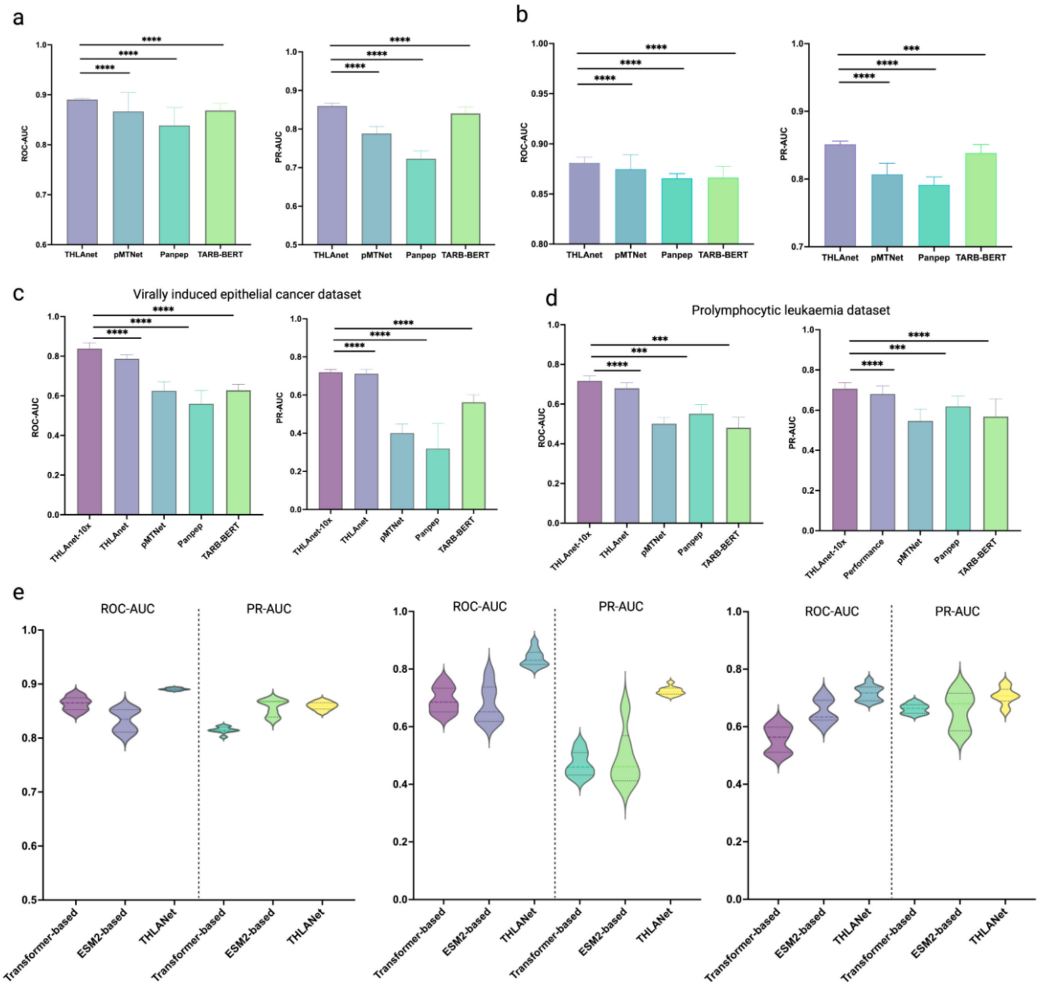

Fig 3. THLANet prediction results. (**a**) ROC-AUC and PR-AUC in the test dataset including 10X Genomics data. (**b**) ROC-AUC and PR-AUC in the test dataset from public databases. (**c**) ROC-AUC and PR-AUC in melanoma and gastrointestinal cancer datasets. (**d**) ROC-AUC and PR-AUC in prolymphocytic leukemia dataset. (**e**) Ablation experiment, ROC-AUC and PR-AUC with two-tailed Wilcoxon signed-rank test adjusted p-values across methods.

Supplementary Table S9. Software and Hardware Environment

File	Parameter Category	Parameter Name	Value/Description
**THLAnet**	**Model Parameters**	kernel sizes	[1, 2, 1]
		hidden_dims	[1024, 512, 256]
		dropout_rate	0.3
		Input Shape	[batch_size, 80, 1000]
		Output Shape	[batch_size, 2]
	**Training Parameters**	batch_size	128
		lr	5×10^{-4}
		classification_threshold	0.05
		Optimizer	Adam
		train_split	0.8
		val_split	0.10
		Test Set Proportion	0.10
**Transformer EncoderLayer**	**Model Parameters**	num_layers	3
		d_model	21
		nhead	3
		dim_feedforward	2048
		dropout	0.1
		batch_size	Default 32
**ESM embedding**	**Model Parameters**	Model Architecture	ESM-2 t33, 650M parameters, UR50D pretrained
		Output Shape	[num_samples, 80, 1280]
**ESM2 pretrain**	**Model Parameters**	Model Architecture	ESM-2 650M, 33 layers
		Freeze Strategy	Freeze all parameters, unfreeze first 25 layers
	**Training Parameters**	num_train_epochs	2
		per_device_train_batch_size	2
		learning_rate	1e-5
		save_steps	500
		Optimizer	AdamW

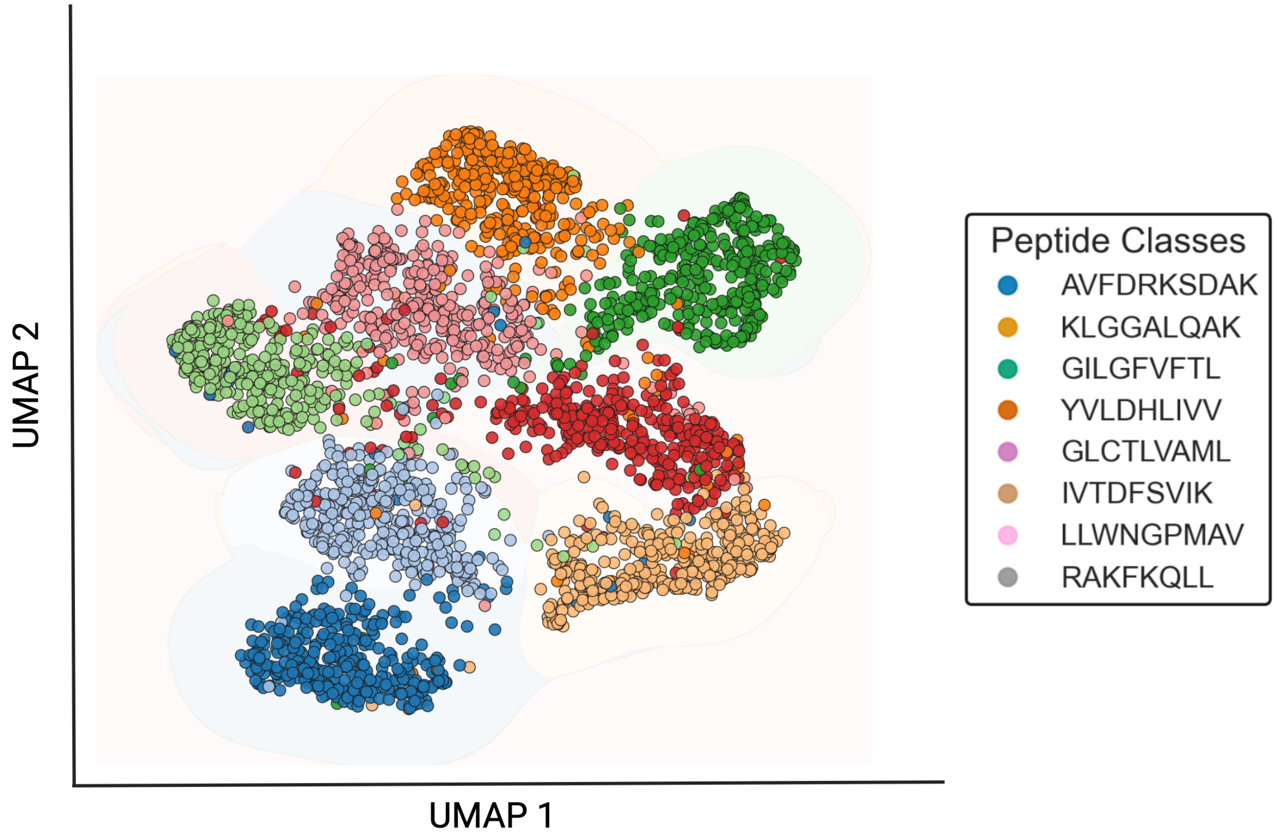

Fig1b. Uniform manifold approximation and projection (UMAP) projections of the predicted epitope-specific TCR clusters.

2.

Comment:

The benchmarking of THLANet appears limited, relying mainly on the authors' curated test dataset. I strongly suggest performing a comprehensive and rigorous benchmark evaluation using standard public datasets (VDJdb and McPAS-TCR) separately, clearly distinguishing training and testing data to demonstrate broader robustness and generalisability.

Reply:

We sincerely thank the reviewers for suggesting the use of standard public datasets (VDJdb and McPAS-TCR) for comprehensive and rigorous benchmarking, as well as clearly distinguishing between training and testing data to demonstrate broader robustness and generalization capabilities. In response to this suggestion, we removed the 10X Genomics data from the original dataset and added human-related

data samples from McPAS-TCR, constructing a new standard baseline dataset comprising VDJdb, IEDB, and McPAS-TCR to showcase enhanced robustness and generalization capabilities. Finally, we constructed a baseline dataset which have 40,748 unique TCR-pHLA pairs.

As shown in Fig.3b, we trained the models on a dataset derived from VDJdb, IEDB, and McPAS-TCR, where THLAnet consistently outperformed other baseline models. Notably, THLAnet achieved the most remarkable performance in the PR-AUC metric, attaining a score of 0.8503, indicating its superior capability in identifying positive samples compared to other baseline models. Meanwhile, we tested the model trained on the benchmark dataset using the cancer dataset and the prolymphocytic leukemia dataset, both achieving results comparable to those of the dataset including 10X Genomics. Detailed test data could be found in Table S10 of Supplementary Materials.

Please find the corresponding revision in the “Data curation” section, lines “104-109” on page 4, the sections of “THLANet outstanding Performance” and “THLANet exhibits potential in multi-cancer clinical applications” in lines “257-323” on pages 8-12.

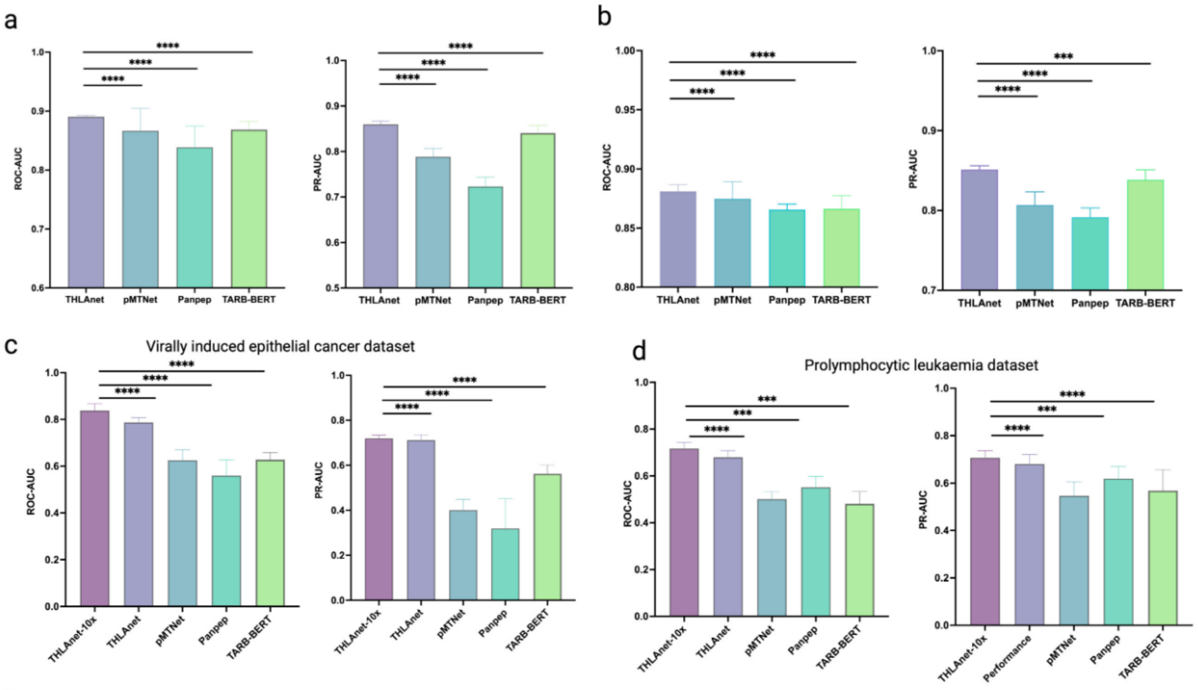

Fig 3. THLANet prediction results. (a) ROC-AUC and PR-AUC in the test dataset including 10X Genomics data. (b) ROC-AUC and PR-AUC in the test dataset from public databases. (c) ROC-AUC and PR-AUC in melanoma and gastrointestinal cancer datasets. (d) ROC-AUC and PR-AUC in prolymphocytic leukemia dataset.

3.

Comment:

Although data dependency is identified by the authors as a limitation in other methods, the current manuscript lacks explicit testing of THLANet's own potential data dependency issues. Additional experiments, such as systematically varying training set sizes, or evaluating performance on completely unseen epitopes or HLAs, are necessary to conclusively address how THLANet handles data dependency.

Reply:

We sincerely appreciate the reviewer's insightful suggestion to explicitly evaluate potential data dependency issues in THLANet. To address this, we conducted additional experiments to assess THLANet's performance on entirely unseen epitopes and human leukocyte antigens (HLAs), systematically varied training set sizes, and employed a dynamic sampling strategy to mitigate data dependency, thereby demonstrating THLANet's robustness and generalizability.

To evaluate THLANet's performance on unseen epitopes and HLAs, we utilized the "COVID-19 dataset" from the ImmuneCODE project, after comparison and cleaning, we got a dataset which have 2,876 unique TCR-pHLA pairs, curated to exclude any epitopes or HLA alleles present in the training set. Without retraining, THLANet achieved a receiver operating characteristic area under the curve (ROC-AUC) of 0.6873 and a precision-recall area under the curve (PR-AUC) of 0.4107, significantly outperforming baseline models: PanPep (ROC-AUC: 0.5987, PR-AUC: 0.2848), pMTNet (ROC-AUC: 0.6056, PR-AUC: 0.3262), and TARB-BERT (ROC-AUC: 0.6077, PR-AUC: 0.3669) (Fig. 4a). These results were underscoring THLANet's generalizability to novel immunological contexts, attributable to its hybrid architecture integrating evolutionary scale modeling-2 (ESM-2) embeddings with a bilinear attention network.

To address the long-tail distribution and data dependency in TCR-pHLA interaction datasets, we implemented an HLA allele-specific sampling strategy during THLAnet's training. This approach dynamically weighted samples based on HLA allele frequency 229 to balance rare and common alleles. By mitigating bias from overrepresented alleles, it enhanced model robustness and generalization.

To systematically investigate the impact of training set size, we retrained THLANet on subsets of the integrated dataset (66,314 TCR-pHLA pairs; Page 3) at 20%, 40%, 60%, 80%, and 100% of the original size, evaluating performance on the unseen dataset. Notably, a sensitivity analysis with the training data reduced to 40% revealed minimal performance degradation (ROC-AUC: ~0.65, PR-AUC: ~0.39; Figure S6 in S1 Text). This highlights THLANet's data efficiency and reduced reliance on large datasets, further affirming its robustness against data dependency.

Please find the corresponding revision in the "Dynamic Sampling Strategy" section, lines "237-242" on page 8, "Superior Performance of THLAnet on Unseen Datasets" in lines "332-347" on pages 12-13.

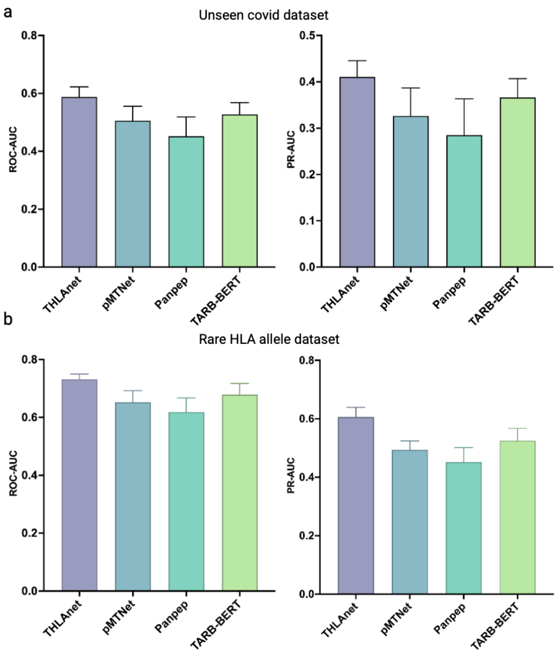

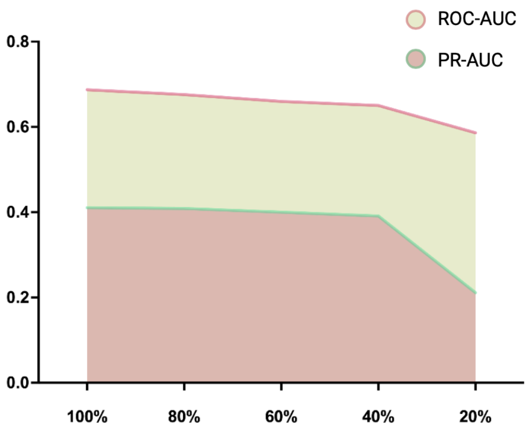

Fig 4. (a) ROC-AUC and PR-AUC for THLAnet, PanPep, pMTnet, and TARB-BERT on the unseen COVID-19 dataset from ImmuneCODE (2876 TCR-pHLA pairs). (b) ROC-AUC and PR-AUC for THLAnet and baseline models on low-frequency HLA class I dataset.

Supplementary Fig. 6. Sensitivity analysis.

4.

Comment:

The clinical relevance of peptides used for training and evaluation is unclear. The manuscript should clearly state their intended role and explicitly acknowledge whether these peptides have direct clinical relevance or serve only as proxies to validate binding prediction capability.

Reply:

Thank you for your insightful comment regarding the clinical relevance of the peptides used for training and evaluation. We fully agree that clarifying their intended role is crucial for transparency and to avoid any ambiguity about their applicability in clinical settings.

To address this concern, we have revised the "Data curation" subsection in the "Materials and methods" section. Specifically, we have added explicit statements explaining that the peptides sourced from databases like VDJdb and IEDB include a mixture of clinically relevant epitopes (e.g., tumor-associated neoantigens

No. 92, West Dazhi Street, Nangang District,
Harbin, Heilongjiang, China.
T : 86-0451-86412114
F : 86-0451-86221048
W: www.hit.edu.cn

from cancers such as melanoma and gastrointestinal tumors) and non-clinical or viral epitopes. We acknowledge that while some peptides have direct clinical relevance in immunotherapy (e.g., neoantigens from somatic mutations in cancer patients), others primarily serve as proxies to validate the model's binding prediction capability, without implying direct therapeutic use. Additionally, in the description of the tumor-mutant immunogenic antigen dataset, we have emphasized their direct clinical relevance as tumor-specific neoantigens that inform immunotherapy strategies, while also noting their role as validation proxies.

Please find the corresponding clarification in the "Data curation" section in lines "75-82" and "122-125" on pages 3-4 and Supplementary Table S11 in the Supplementary Material.

Supplementary Table S11. The list of pMHC multimers used by 10x Genomics

Allele	Category	Peptide
A*0101	IE-1/CMV	VTEHDTLLY
A*0201	gp100/Cancer	KTWGQYWQV
A*0201	MART-1/Cancer	ELAGIGILTV
A*0201	Tyrosinase/Cancer	CLIWSFQTSA
A*0201	gp100/Cancer	IMDQVPFSV
A*0201	NY-ESO-1/Cancer	SLLMWITQV
A*0201	MAGE A3/Cancer	KVAELVHFL
A*0201	MAGE-A1/Cancer	KVLEYVIKV
A*0201	Kanamycin B dioxygenase	CLLGTYTQDV
A*0201	EBNA 3B/EBV	LLDFVRFMGV
A*0201	HPV 16E7, 82-91	LLMGTLGIVC
A*0201	LMP-2A/EBV	CLGGLITMV
A*0201	LMP1/EBV	YLLEMLWRL
A*0201	LMP2A/EBV	FLYALALLL
A*0201	Flu MP/Influenza	GILGFVFTL
A*0201	BMLF1/EBV	GLCTLVAML
A*0201	pp65/CMV	NLVPMVATV
A*0201	RT/HIV	ILKEPVHGV
A*0201	Ca2+-indepen. Plip A2	FLASKIGRLV
A*2402	WT1 (235-243)236M->Y	CYTWNQMNL
A*0201	Gag protein/HIV	RTLNAWVKV
A*0201	PSA 146-154	KLQCVDLHV
A*0201	HTLV-1	LLFGYPVYV
A*0201	Gag protein/HIV	SLYNTVATL
A*0201	Gag protein/HIV	SLYNTVATLY
A*0201	Gag protein/HIV	SLENTVATLY
A*0201	WT-1	RMFPNAPYL
A*0201	BCL-X/Cancer	YLNDHLEPWI
A*0201	16E7/HPV	MLDLQPETT
A*0301	IE-1/CMV	KLGGALQAK
A*0301	EBNA 3A/EBV	RLRAEAQVK
A*0301	BCL-2L1/Cancer	RIAAWMATY
A*1101	EBNA 3B/EBV	IVTDFSVIK
A*1101	EBNA 3B/EBV	AVFDRKSDAK
B*3501	pp65/CMV	IPSINVHHY
A*2402	IE-1/CMV	AYAQKIFKI
A*2402	pp65/CMV	QYDPVAALE
B*0702	EBNA 6/EBV	QPRAPIRPI
B*0702	pp65/CMV	TPRVTGGGAM
B*0702	EBNA 3A/EBV	RPPIFIRRL
B*0702	pp65/CMV	RPHERNGFTVL
B*0801	BZLF1/EBV	RAKFKQLL
B*0801	IE-1/CMV	ELRRKMMVM
B*0801	EBNA 3A/EBV	FLRGRAYGL

No. 92, West Dazhi Street, Nangang District,
Harbin, Heilongjiang, China.
T : 86-0451-86412114
F : 86-0451-86221048
W: www.hit.edu.cn

5.

Comment:

Typically, neoantigen discovery pipelines begin with the filtering of peptides based on their ability to bind MHC molecules. The manuscript does not clarify if peptides included in the study are experimentally validated MHC binders or predicted binders. Clarifying this point explicitly would address potential concerns about the biological validity of negative samples and their potential impact on model performance.

Reply:

We sincerely thank the reviewer for their insightful comment regarding the validation status of peptides used in our study and their impact on model performance.

To address this, we have revised the “Data curation” section to explicitly clarify that all peptides included in our study are experimentally validated MHC binders. Specifically, the datasets, sourced from 10x Genomics, VDJdb, and IEDB, consist of laboratory-validated TCR-pMHC interactions, with positive and negative samples confirmed through experimental assays (e.g., single-cell immunoassays and binding assays). Negative samples were selected from validated non-binding TCR-pMHC pairs in IEDB and VDJdb (confidence score of 0), ensuring biological validity. This approach minimizes concerns about false negatives and enhances the reliability of our training dataset, thereby improving THLANet’s predictive performance.

Please find the corresponding clarification in the “Data curation” section in lines “73-88” on pages 3-4.

6.

Comment:

Regarding novelty, the manuscript utilises common deep-learning building blocks such as the ESM-2 language model and transformer architectures, making the novelty somewhat incremental. To justify claims of innovation, the manuscript should clearly articulate what distinct methodological contributions or novel applications THLANet provides compared to existing state-of-the-art approaches.

Reply:

We sincerely thank the reviewer for their insightful comment regarding the novelty of THLANet and the need to articulate its distinct contributions compared to state-of-the-art approaches.

To address this, we have revised the “Introduction” and “Discussion” sections to explicitly highlight THLANet’s methodological innovations and novel applications. Specifically, THLANet introduces a hybrid architecture that uniquely integrates ESM-2 embeddings with a bilinear attention network and sinusoidal positional encoding via a Transformer-Encoder, enabling robust capture of TCR–pHLA

No. 92, West Dazhi Street, Nangang District,
Harbin, Heilongjiang, China.
T : 86-0451-86412114
F : 86-0451-86221048
W: www.hit.edu.cn

interaction nuances. Unlike PanPep, which relies on meta-learning and neural Turing machines for task adaptation, THLANet employs a segment-based CDR3 analysis to identify critical binding residues, validated against PDB crystal structures, enhancing interpretability for immunotherapy design. Compared to pMTnet, which uses stacked autoencoders and LSTM, THLANet's bilinear attention mechanism better fuses TCR and pHLA embeddings, improving prediction accuracy for diverse datasets. In contrast to TABR-BERT, which separates TCR and pMHC encoding, THLANet's unified encoding strategy minimizes information loss, achieving superior generalization for unseen epitopes. Additionally, THLANet's application to alanine-scanning mutagenesis provides predictive structural insights, directly supporting neoantigen vaccine optimization and TCR-T therapy.

These revisions emphasize THLANet's unique contributions in architecture design and clinical applicability. Please find the corresponding updates in the "Introduction" section in lines "43-54" on page 3 and the "Discussion" section in lines "448-462" on pages 15-16.

7.

Comment:

The dataset shows significant biases towards a limited set of HLA alleles (mostly HLA-A), with underrepresentation of other HLA class I alleles. This raises questions about THLANet's general applicability. The manuscript needs to demonstrate explicitly whether the model's performance remains consistent across diverse HLA class I alleles and discuss the implications of potential performance drops for rare alleles.

Reply:

We sincerely appreciate the reviewer's concern regarding the potential bias in our dataset toward a limited set of HLA alleles, particularly HLA-A, and the necessity to demonstrate THLANet's consistent performance across diverse HLA class I alleles. To address this, we clarify that our dataset is not exclusively biased toward HLA-A and provide additional evidence of THLANet's robust performance across diverse and rare HLA alleles, while also discussing the potential implications of performance variations for rare alleles.

Contrary to the perceived bias, our integrated dataset (66,314 TCR-pHLA pairs; Page 3) encompasses a broad spectrum of HLA class I alleles, including HLA-A, HLA-B, and HLA-C, sourced from VDJdb, IEDB, and 10X Genomics. Specifically, the dataset comprises 1,257 distinct pHLA types, with HLA-B and HLA-C alleles adequately represented alongside HLA-A. To mitigate potential biases, we implemented the HLA allele-specific dynamic sampling strategy described in the manuscript. This approach dynamically weights samples based on HLA allele frequencies, thereby reducing bias toward overrepresented alleles such as HLA-A.

To explicitly evaluate THLANet's generalizability to low-frequency HLA class I alleles, we curated a dataset of 343 laboratory-validated TCR-pHLA interactions involving HLA-B27:05, HLA-B15:01, and HLA-A*24:02 from peer-reviewed studies. These alleles were intentionally selected due to their

No. 92, West Dazhi Street, Nangang District,
Harbin, Heilongjiang, China.
T : 86-0451-86412114
F : 86-0451-86221048
W: www.hit.edu.cn

underrepresentation in the training data. Sequences were processed using ESM-2 and Transformer-Encoder modules, with features integrated via a bilinear attention network, consistent with our primary pipeline. THLANet achieved a mean ROC-AUC of 0.7320 and PR-AUC of 0.6062 (Fig. 4b), significantly outperforming baseline models: pMTNet (ROC-AUC: 0.6527, PR-AUC: 0.4943), PanPep (ROC-AUC: 0.6184, PR-AUC: 0.4521), and TARB-BERT (ROC-AUC: 0.6785, PR-AUC: 0.5252). These results underscore THLANet's robust performance across diverse and rare HLA alleles.

These revisions emphasize THLANet's unique contributions in architecture design and clinical applicability. Please find the corresponding updates in the "Dynamic Sampling Strategy" section in lines "226-231" on page 8, the "Evaluation of THLAnet on Low-Frequency HLA Class I Alleles" in lines "350-363" on page 13 and the "Discussion" section in lines "340-353" on page 15.

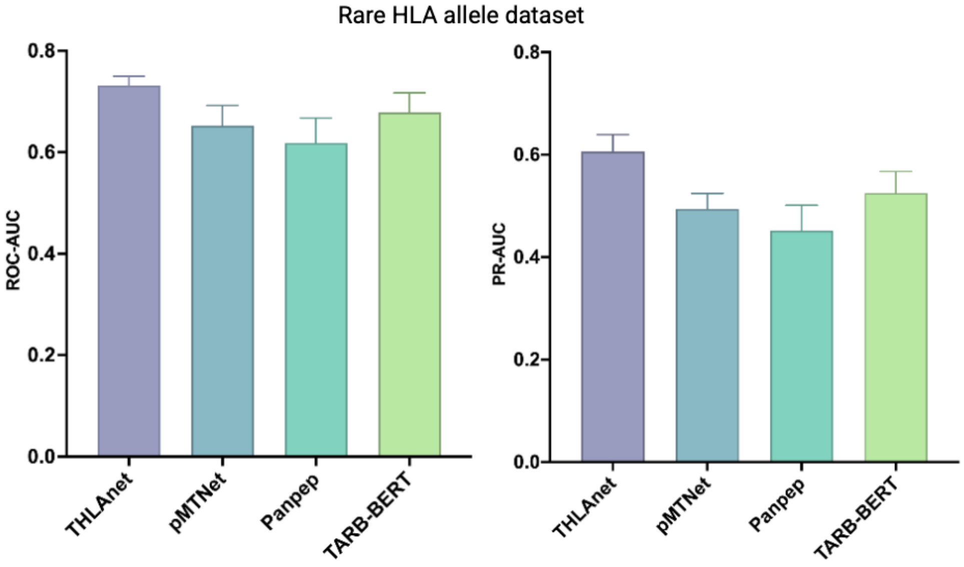

Fig 4b. ROC-AUC and PR-AUC for THLAnet and baseline models on low-frequency HLA class I dataset.

8.

Comment:

The manuscript claims to "unveil 3D binding conformations" of TCR–pHLA complexes, but the evidence provided relies solely on in silico mutational analysis. While computational alanine-scanning experiments are a useful addition, this claim should be toned down to avoid overstating the findings. The authors essentially identify predicted critical residues in TCR and antigen interactions, correlating these predictions with known structural contacts in limited examples. This analysis provides predictive structural insights but does not determine or "unveil" actual 3D conformations. The authors should rephrase such statements to clearly reflect that these are predictive insights. Additionally, more explicit explanation of the method used for dividing CDR3 sequences into segments (beyond figure captions) is required in the main text.

Reply:

No. 92, West Dazhi Street, Nangang District,
Harbin, Heilongjiang, China.
T : 86-0451-86412114
F : 86-0451-86221048
W: www.hit.edu.cn

We sincerely thank the reviewer for their insightful feedback regarding the phrasing of our claims about TCR–pHLA binding conformations and the need for a clearer explanation of CDR3 sequence segmentation.

To address the first point, we have revised the "THLANet provides explanation for the spatial structure of the TCR-pHLA interaction" section to moderate claims about "unveiling 3D binding conformations." The revised text now states that THLANet provides "predictive structural insights" into TCR–pHLA interactions based on in silico alanine-scanning mutagenesis, which identifies critical residues correlated with known structural contacts. This rephrasing clarifies that our findings are predictive rather than definitive determinations of 3D conformations, aligning with the computational nature of the analysis.

For the second point, we have added a detailed description in the THLANet provides explanation for the spatial structure of the TCR-pHLA interaction" section, explicitly outlining the method for dividing CDR3 sequences into segments. Specifically, CDR3 sequences were evenly divided into five segments, with segment lengths dynamically adjusted based on total sequence length (10–25 residues) using a sliding window approach to approximate equal segments, prioritizing the central segment for key contact regions. This method was validated against VDJdb structural annotations to ensure consistency with TCR–pHLA contact points.

Please find the corresponding revisions in the "THLANet provides explanation for the spatial structure of the TCR-pHLA interaction" section in lines "403-410" and "425-432" on page 15.

Minor comments:

1.

Comment:

I found some sentences are extremely difficult to follow (For example, lines 6-8). The manuscript would benefit from significant editing for clarity.

Reply:

We sincerely thank the reviewer for their valuable feedback regarding the clarity of our manuscript.

To address this, we have conducted a thorough review of the entire manuscript, carefully revising sentences that were difficult to follow.

2.

Comment:

No IRB for the blood collection from the healthy donors?

Reply:

哈爾濱工業大學
HARBIN INSTITUTE OF TECHNOLOGY
No. 92, West Dazhi Street, Nangang District,
Harbin, Heilongjiang, China.
T : 86-0451-86412114
F : 86-0451-86221048
W: www.hit.edu.cn

We sincerely thank the reviewer for raising the question regarding Institutional Review Board (IRB) approval for blood collection from healthy donors.

To address this, we clarify in the revised "Data curation" section that our study utilized publicly available datasets provided by 10x Genomics, accessible at https://www.10xgenomics.com/datasets. These open-source datasets, derived from four healthy donors' $CD8^+$ T cells, are freely available for research use and do not involve direct blood collection by our team. Consequently, our experiments did not require IRB approval, as no primary human sample collection was conducted. This ensures compliance with ethical standards while leveraging validated, open-access data for TCR-pHLA analysis.

Data source can be referred to the section "Data Availability" in lines 495-500 (https://www.10xgenomics.com/datasets).

3.

Comment:

No code availability?

Reply:

We sincerely thank the reviewer for highlighting the need for code availability to enhance the reproducibility of our work.

To address this, we have included a "Data Availability" section in the manuscript, which details the availability of the THLANet codebase. Specifically, we have made the code publicly accessible on GitHub at https://github.com/ChanganMakeYi/THLAnet. This section also provides information on the datasets used, sourced from public databases such as IEDB, VDJdb, McPAS-TCR, and 10x Genomics, ensuring transparency and enabling the scientific community to replicate our experiments.

Please find the corresponding revision in the "Data Availability" section in lines "495-500" on page 16.

4.

Comment:

No funding information?

Reply:

We sincerely thank the reviewer for pointing out the absence of funding information.

To address this, we have added a dedicated "Funding" section to the manuscript, detailing the financial support received for this study. This section includes information on the funding sources that supported the development and execution of the THLANet framework, ensuring transparency and

No. 92, West Dazhi Street, Nangang District,
Harbin, Heilongjiang, China.
T : 86-0451-86412114
F : 86-0451-86221048
W: www.hit.edu.cn

compliance with publication requirements.

Please find the corresponding revision in the “Funding” section in lines “484-492” on page 16.
